# Acute inhalation injury caused by chlorine gas exposure

**DOI:** 10.36416/1806-3756/e20260085

**Published:** 2026-05-22

**Authors:** Luciana Volpon Soares Souza, Arthur Soares Souza, Edson Marchiori

**Affiliations:** 1. Ultra X, São José do Rio Preto (SP) Brasil.; 2. Faculdade de Medicina de Rio Preto, São José do Rio Preto (SP) Brasil.; 3. Universidade Federal do Rio de Janeiro, Rio de Janeiro (RJ) Brasil.

**Figure 1:**
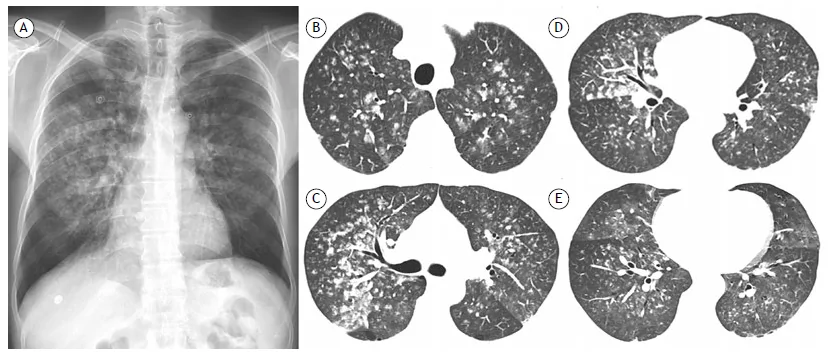
Chest radiograph (A) showing non-homogeneous opacities, predominantly in the middle thirds of the lungs. Axial chest CT images (B-D) showing ground-glass nodules and areas of consolidation and ground-glass attenuation, distributed diffusely throughout the lungs.

A 48-year-old man, employed as an outdoor swimming pool cleaner, sought medical attention after acute exposure to chlorine at work. Immediately after inhalation of the gas, he developed cough and dyspnea, which progressively worsened throughout the day, without fever, sputum, or hemoptysis. His blood pressure was 90/60 mmHg, his respiratory rate was 25 bpm, and his oxygen saturation was 95%. Laboratory test results were normal. Chest radiography and chest CT showed ground-glass nodules, areas of consolidation, and ground-glass attenuation (Figure 1). Bronchoscopy with bronchoalveolar lavage yielded negative results for infectious agents. Oxygen therapy, intravenous hydrocortisone, and nebulization were initiated, and the patient was hospitalized with a provisional diagnosis of chemical pneumonitis. He showed progressive improvement and was discharged for outpatient follow-up, with complete pulmonary function recovery. A follow-up CT scan performed one month later was normal.

Chlorine, a dense and irritating gas, is commonly used worldwide in factories and many consumer products, such as insecticides and disinfectants. Its accidental inhalation occurs in household and industrial contexts and can lead to severe respiratory complications, including airway damage, alveolar injury, pulmonary edema, and ARDS. ARDS secondary to chlorine gas inhalation is a rare but serious complication, often requiring intensive care.^1^ ^-^ ^3^ Chlorine exposure also has long-term effects such as bronchitis, bronchiolitis obliterans, and emphysema.^1^ ^,^ ^3^ There is no specific treatment for acute injuries caused by chlorine gas inhalation; management is mainly supportive.
